# Involvement of Autophagy in Levodopa‐Induced Dyskinesia

**DOI:** 10.1002/mds.28480

**Published:** 2021-01-18

**Authors:** Michael Feyder, Carina Plewnia, Ori J. Lieberman, Giada Spigolon, Alessandro Piccin, Lidia Urbina, Benjamin Dehay, Qin Li, Per Nilsson, Mikael Altun, Emanuela Santini, David Sulzer, Erwan Bezard, Anders Borgkvist, Gilberto Fisone

**Affiliations:** ^1^ Department of Neuroscience Karolinska Institutet Stockholm Sweden; ^2^ Departments of Neurology, Pharmacology and Psychiatry Columbia University, and New York State Psychiatric Institute New York New York USA; ^3^ Univ. Bordeaux, CNRS, IMN, UMR 5293 Bordeaux F‐33000 France; ^4^ Motac Neuroscience Ltd Manchester United Kingdom; ^5^ Institute of Laboratory Animal Sciences & China Academy of Medical Sciences Beijing China; ^6^ Department of Neurobiology, Care Sciences and Society, Center for Alzheimer Research, Division of Neurogeriatrics Karolinska Institutet Stockholm Sweden; ^7^ Science for Life Laboratory, Department of Laboratory Medicine Karolinska Institutet, Karolinska University Hospital Stockholm Sweden

**Keywords:** Parkinson's disease, l‐dopa, p62, autophagy, striatum

## Abstract

**Background:**

Autophagy is intensively studied in cancer, metabolic and neurodegenerative diseases, but little is known about its role in pathological conditions linked to altered neurotransmission. We examined the involvement of autophagy in levodopa (l‐dopa)‐induced dyskinesia, a frequent motor complication developed in response to standard dopamine replacement therapy in parkinsonian patients.

**Methods:**

We used mouse and non‐human primate models of Parkinson's disease to examine changes in autophagy associated with chronic l‐dopa administration and to establish a causative link between impaired autophagy and dyskinesia.

**Results:**

We found that l‐dopa‐induced dyskinesia is associated with accumulation of the autophagy‐specific substrate p62, a marker of autophagy deficiency. Increased p62 was observed in a subset of projection neurons located in the striatum and depended on l‐dopa‐mediated activation of dopamine D1 receptors, and mammalian target of rapamycin. Inhibition of mammalian target of rapamycin complex 1 with rapamycin counteracted the impairment of autophagy produced by l‐dopa, and reduced dyskinesia. The anti‐dyskinetic effect of rapamycin was lost when autophagy was constitutively suppressed in D1 receptor‐expressing striatal neurons, through inactivation of the autophagy‐related gene protein 7.

**Conclusions:**

These findings indicate that augmented responsiveness at D1 receptors leads to dysregulated autophagy, and results in the emergence of l‐dopa‐induced dyskinesia. They further suggest the enhancement of autophagy as a therapeutic strategy against dyskinesia. © 2021 The Authors. *Movement Disorders* published by Wiley Periodicals LLC on behalf of International Parkinson and Movement Disorder Society

## Graphical abstract

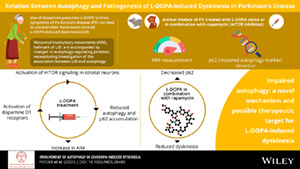

May Infographic: Involvement of Autophagy in Levodopa‐Induced Dyskinesia

Disruption of dopamine transmission is a cardinal feature of Parkinson's disease (PD), a common neurodegenerative disorder characterized by severe motor impairment. PD motor symptoms are effectively treated with the dopamine precursor levodopa (l‐dopa), but the use of this drug is often limited by the appearance of dystonic and choreic motor complications referred to as l‐dopa‐induced dyskinesia (LID).^1^

A critical factor in the emergence of LID is the development of sensitization at dopamine D1 receptors (D1Rs) located in the dorsal striatum. This phenomenon, which has been observed in postmortem samples from parkinsonian patients^2^ and in experimental models of PD,^3^, ^4^, ^5^ has been interpreted as an adaptive response to compensate for the loss of dopamine input to the basal ganglia.^5^ The enhanced sensitivity of D1Rs, which are selectively expressed in the striatal projection neurons of the direct pathway (dSPN), potentiates the action of l‐dopa and leads to the stimulation of multiple signaling pathways, including the mammalian target of rapamycin (mTOR) cascade.^6^, ^7^ Increased mTOR signaling in dSPN is a major culprit behind the development of LID. Thus, administration of rapamycin, a selective inhibitor of the mTOR complex 1 (mTORC1), reduces abnormal involuntary movements (AIMs), a surrogate marker of LID, in rodent models of PD.^6^, ^8^ mTORC1 plays a key role in the control of protein synthesis, via activation of downstream effector targets that promote initiation of translation and elongation of mRNA,^9^ as well as upregulation of ribosomal proteins and translation factors.^10^

Another essential role of mTORC1 pertains to its ability to regulate autophagy, an intracellular pathway involved in lysosomal degradation of protein aggregates and pathogens, as well as in cellular processes such as phagocytosis, secretion, and exocytosis.^11^ Activation of mTORC1 inhibits autophagy through phosphorylation of the Unc‐51like kinase 1 (Ulk1), a mammalian homolog of the autophagy‐related gene protein (Atg) 1 originally described in yeast.^12^ mTORC1‐mediated phosphorylation at S757 prevents Ulk1 forming a core complex with other Atg required for the generation of the autophagosome.^13^, ^14^ Changes in autophagy can be monitored by measuring the levels of the specific substrate p62 (also named sequestosome 1). During autophagy, p62 interacts with polyubiquitinated proteins and is targeted to the autophagosome, where it is eliminated together with its cargo by lysosomal degradation.^15^, ^16^ Accumulation of p62 is therefore regarded as a marker of impaired autophagy.^15^, ^16^

Inhibition of mTORC1 has been shown to reduce dyskinetic behavior in experimental models of PD,^6^, ^17^, ^18^, ^19^ however the mechanisms at the basis of this effect remain to be established. In this study, we show that LID is associated with impaired autophagy and that rapamycin exerts an anti‐dyskinetic effect by counteracting this condition.

## Materials and Methods

### Animals

C57BL/6J mice (25–30 g) were purchased from Charles River (Sulzfeld, Germany). Mice expressing enhanced green fluorescent protein (EGFP) or Cre recombinase under the control of the promoter for the D1R [*Drd1a‐*EGFP mice, *Drd1a‐*Cre (EY262)] were generated by the GENSAT (Gene Expression Nervous System Atlas) program at the Rockefeller University^20^ and were crossed on a C57BL/6 background for at least 10 generations. Conditional knockout mice of Atg7 in D1R‐expressing SPN (*Atg7* ^*F/F*^ *;Drd1a‐Cre* ^*+/−*^ mice) and control mice (*Atg7* ^*F/F*^) were from the offspring of *Atg7* ^*F/F*^ (gift from Masaaki Komatsu, Juntendo University School of Medicine, Japan) and *Atg7* ^*F/F*^ *;Drd1a‐Cre* ^*+/−*^ mice. Experiments were carried out in accordance with the guidelines of the Research Ethics Committee of Karolinska Institutet, Swedish Animal Welfare Agency, and European Communities Council Directive 86/609/EEC. Captive‐bred female monkeys (*Macaca mulatta*; Xierin, Beijing) were housed in individual cages under controlled conditions of humidity, temperature, and light with food and water ad libitum. Animal care was supervised by veterinarians skilled in healthcare and maintenance. Experiments were carried out in accordance with European Communities Council Directive of 3 June 2010 (2010/6106/EU) for care of laboratory animals, in an Association for Assessment and Accreditation of Laboratory Animal Care accredited facility. Procedures were approved by the Institute of Laboratory Animal Science ethical committee.

### Drugs

6‐Hydroxydopamine‐HCl (6‐OHDA; Sigma‐Aldrich Sweden AB, Stockholm, Sweden) was dissolved in saline containing 0.02% ascorbic acid. l‐dopa and benserazide hydrochloride (Sigma‐Aldrich Sweden AB), and SCH23390 and raclopride (Tocris‐Biotechne Ltd., Abingdon, UK) were dissolved in saline and injected intraperitoneally (IP) in a volume of 10 ml/kg body weight. Rapamycin (LC Laboratories, Woburn, MA) was dissolved in 5% dimethyl sulfoxide, 5% Tween‐20, 15% polyethylene glycol, and distilled water, and administered IP in a volume of 2 ml/kg body weight.

### 6‐OHDA Lesion and LID in Mice

Mice were injected subcutaneously with Temgesic as analgesic, and positioned in a stereotaxic frame (David Kopf Instruments, Tujunga, CA). Anesthesia was induced with 4% isoflurane and maintained with 2% isoflurane. Each mouse was injected with 1 μL of vehicle containing 3.75 μg of free‐base 6‐OHDA into the right medial forebrain bundle, according to the following coordinates (millimeters, relative to bregma): anteroposterior (AP), −1.2; mediolateral (ML), −1.2; dorsoventral (DV), −4.8.^21^ The needle was left in place for 5 minutes before and after injection. Mice were allowed to recover for 3 weeks before experimentation. Only animals with a tyrosine hydroxylase (TH, a marker of dopamine terminals) reduction of 90% or more in the dorsal striatum were included in the study. *Atg7* ^*F/F*^ *;Drd1a‐*Cre^*+/−*^ mice and *Atg7* ^*F/F*^ littermates with a unilateral 6‐OHDA lesion were treated for 9 days with 10 mg/kg of l‐dopa, administered alone or in combination with rapamycin (5 mg/kg). AIMs were assessed after the last injection using a previously established scale.^22^ Briefly, 20 minutes after the last injection, mice were placed in separated cages and individual dyskinetic behaviors (ie, AIMs) were assessed for 1 minute every 20 minutes, over a period of 120 minutes. AIMs were classified into four subtypes: locomotive AIMs (contralateral turns), axial AIMs (dystonic posturing of the upper part of the body toward the side contralateral to the lesion), limb AIMs (abnormal movements of the forelimb contralateral to the lesion), and orofacial AIMs (vacuous jaw movements and tongue protrusion). Each subtype was scored on a severity scale from 0 to 4: 0, absent; 1, occasional; 2, frequent; 3, continuous; 4, continuous and not interruptible by outer stimuli.

### MPTP Lesion and LID in Monkeys

PD modeling in non‐human primates (NHP) and tissue collection is based on a previously used and described experimental cohort. MPTP (1‐methyl‐4‐phenyl‐1,2,3,6‐tetrahydropyridine) intoxication protocol, chronic l‐dopa treatment, the clinical assessments, the terminal procedure, and the characterization of the extent of nigrostriatal denervation were conducted as previously published.^19^, ^23^, ^24^, ^25^ Briefly, macaques received daily saline or MPTP hydrochloride injections (0.2 mg/kg, intravenously) until parkinsonian signs appeared. Once PD motor signs were stable, MPTP‐treated monkeys were either untreated or treated twice a day with an individually titrated dose of l‐dopa (Modopar, l‐dopa/carbidopa, 4:1 ratio; range, 9–17 mg/kg). This dose, defined as 100% dose, was used for chronic l‐dopa treatment, which lasted for 4 to 5 months until dyskinesia stabilized. A nigrostriatal lesion above 95% was reported in both MPTP groups in comparison to control animals as previously reported.^26^ Brain patches collected from 300 μm‐thick fresh frozen coronal sections containing caudate‐putamen were collected for Western blotting analysis as previously reported.^25^

### Tissue Preparation and Western Blotting

Mice were killed by decapitation, punches of striatal tissue (1 mm thickness, 2 mm diameter; three punches per hemisphere) were dissected, sonicated in 1% SDS and boiled for 10 minutes. Proteins/samples (30 μg) were loaded onto 10% polyacrylamide gels and separated by electrophoresis and transferred overnight to polyvinylidene fluoride (PVDF) membranes (Amersham Pharmacia Biotech, Uppsala, Sweden).^27^ The membranes were immunoblotted with antibodies against p62 (1:1000, Abcam), Ulk1, phospho‐S757‐Ulk1, S6 and phospho‐S240/244‐S6 (1:1000, Cell Signaling Technology, Leiden, The Netherlands), actin (1:30000, Sigma‐Aldrich Sweden AB), and TH (1:1000, Millipore). Detection was based on fluorescent secondary antibody binding and quantified using a Li‐Cor Odyssey infrared fluorescent detection system (Li‐Cor, Lincoln, NE). The levels of phospho‐S757‐Ulk1 and phospho‐S240/244‐S6 were normalized according to the levels of the respective total protein. Monkey tissue patches were extracted on ice and placed in 100 μl of RIPA buffer (50 mM Tris–HCl pH 7.4, 150 mM NaCl, 1.0% Triton X‐100, 0.5% Na‐deoxycholate, 0.1% sodium dodecyl sulfate) with a protease inhibitor cocktail tablet (Complete Mini, Roche Diagnostics). The lysate was incubated on ice for 20 minutes and centrifuged at 14,000 rpm for 15 minutes at 4°C. The supernatant was collected and stored at −80°C. Proteins/samples (20 μg) were separated by sodium dodecylsulfate‐polyacrylamide gel electrophoresis and transferred to nitrocellulose membranes. Incubation with primary antibodies was performed overnight at 4°C with antibodies against p62 (1:1000, Progen) and actin (1:5000, Sigma). Appropriate secondary antibodies coupled to peroxidase were revealed using a Super Signal West Pico Chemiluminescent kit (Immobilon Western, Chemiluminescent HRP substrate, Millipore). Chemiluminescence images were acquired using the ChemiDoc+XRS system measurement (BioRad). Signals per lane were quantified using ImageJ and a ratio of signal on loading per animal was performed and used in statistical analyses.

### Immunofluorescence

Mice with the unilateral 6‐OHDA lesion were deeply anesthetized with sodium pentobarbital (100 mg/kg, IP, Sanofi‐Aventis, France) and perfused transcardially with 4% (weight/vol) ice‐cold paraformaldehyde in 0.1 M phosphate buffer. The brains were post‐fixed overnight in the same solution and 40 μm‐thick coronal sections were cut with a vibratome (Leica, Germany). Triple‐labeling for p62/DARPP‐32/EGFP was performed as follows. Sections (each one containing an intact and a 6‐OHDA lesion striatum) were washed in Tris‐buffered saline solution (TBS) (100 mM Tris‐Cl, 150 mM NaCl, pH 7.5), incubated for 1 hour at room temperature in 1% bovine serum albumin (BSA)‐0.3% Triton X‐100‐TBS, and then kept overnight at 4°C in 1% BSA‐TBS solution containing chicken EGFP (1:1000, GFP‐1020, Aves Labs), rabbit p62 (1:500, ab91526, Abcam), and mouse DARPP‐32 (1:1000) antibodies. After TBS washing, sections were incubated for 1 hour at room temperature in 1% BSA‐TBS solution containing Alexa Fluor‐647 anti‐mouse, Alexa Fluor‐488 anti‐chicken, and Cy3 anti‐rabbit secondary antibodies (1:400). Z‐stack images of the dorsal striatum were captured using sequential laser scanning confocal microscopy (Zeiss LSM 8, Carl Zeiss, Germany) at 63× magnification. Acquired images were used to quantify p62 and EGFP immunofluorescence in *Drd1a‐*EGFP mice with the open‐source analysis platform Fiji.^28^ Displayed gray value was set to 0–4082 for all images and p62+/EGFP+ and p62+/EGFP− cells were counted in the 6‐OHDA lesion and control striatum (n = 5 mice).

### p62 RNA Expression Analysis

One striatal punch per hemisphere was dissected out and snap‐frozen in liquid nitrogen for further processing. Total RNA was extracted using a RNeasy kit (Qiagen) and quantified on a NanoDrop 1000 device. RNA (200 ng) was used for generation of cDNA using iScript cDNA Synthesis Kit (Bio‐Rad) and iTaq Universal SYBR Green Supermix (Bio‐Rad) on a CFX96 Touch Real‐Time PCR Detection System (Bio‐Rad) with primers Sqstm1 (5′‐TTCGGAAGTCAGCAAACCTGA‐3′ and 5′‐CCGACTCCATCTGTTCCTCTG‐3′) and GAPDH (5′‐AGGTCGGTGTGAACGGATTTG‐3′ and 5′‐TGTAGACCATGTAGTTGAGGTCA‐3′) for gene expression.

### Statistical Analyses

Data are presented as mean ± SEM. Differences between groups were evaluated by one‐ or two‐way ANOVA with post hoc multiple comparison test as described, while two‐group comparisons were performed with Welch two‐sample or unpaired Student's *t*‐test.

## Results

**FIG. 1:**
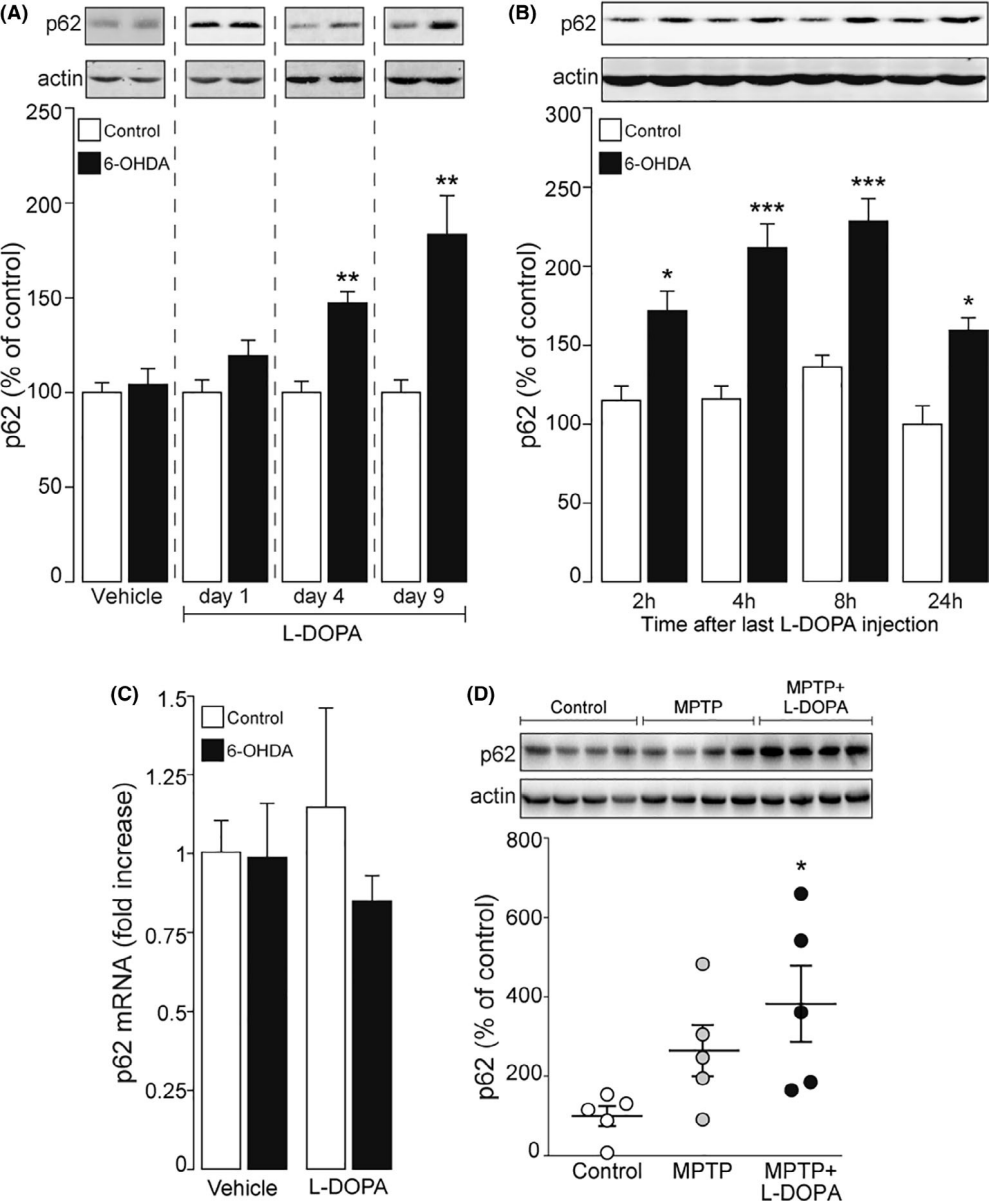
Chronic administration of levodopa (l‐dopa) reduces autophagy in the striata of parkinsonian mice and non‐human primates. C57BL/6 mice with a unilateral 6‐hydroxydopamine (6‐OHDA) lesion were treated with l‐dopa (10 mg/kg) as described, and the levels of p62 were measured by Western blotting in the striata contralateral (control) or ipsilateral (6‐OHDA) to the lesion. (**A**) Mice were divided in four groups (n = 4–10) and treated with vehicle, or with l‐dopa for 1, 4, and 9 days, and p62 was measured 4 hours after the last injection. **P < 0.01 versus control, Welch two‐sample *t*‐test. (**B**) Four groups (n = 6–7) of mice were treated with l‐dopa for 9 days and p62 was measured 2, 4, 8, or 24 hours after the last injection. Two‐way ANOVA showed a significant effect of 6‐OHDA lesion (F_1,45_ = 86.01, *P* < 0.001) and a significant effect of time (F_3,45_ = 8.16, *P* < 0.001), but no lesion × time interaction (F_3,45_ = 1.61, *P* = 0.199). *P < 0.05 and ***P < 0.001 versus control, Tukey post hoc test. (**C**) Two groups (n = 6–9) of mice were treated with vehicle or l‐dopa for 9 days and mRNA for p62 was measured 4 hours after the last injection. (**D**) p62 was measured in the striata of three experimental groups (n = 5) of non‐human primates: normal (control), parkinsonian (1‐methyl‐4‐phenyl‐1,2,3,6‐tetrahydropyridine; MPTP), and parkinsonian treated with l‐dopa (MPTP+l‐dopa). *P < 0.05 versus control group, one‐way ANOVA and Tukey's multiple comparison test.

In mice with a unilateral 6‐OHDA lesion, repeated daily administration of l‐dopa (10 mg/kg), which induces severe AIMs,^6^ increased the levels of p62 in the striatum ipsilateral to the 6‐OHDA lesion compared to the intact (control) contralateral striatum (Fig. 1A, days 4 and 9). In contrast, a single administration of l‐dopa (Fig. 1A, day 1) did not produce any effect. The increase in p62 caused by chronic l‐dopa in the dopamine denervated striatum peaked at 8 hours and persisted for up to 24 hours after the last drug administration (Fig. 1B). Increased p62 protein levels were not paralleled by enhanced p62 mRNA (Fig. 1C), indicating that the effect of l‐dopa was exerted via reduced degradation, rather than augmented transcription. In line with the results obtained in the mouse, p62 was significantly increased also in the gold standard NHP model of LID based on MPTP intoxication^19^, ^23^, ^24^, ^25^ (Fig. 1D). Altogether, these results indicated that chronic treatment with l‐dopa leads to impaired autophagy in the dopamine‐depleted striatum.

**FIG. 2:**
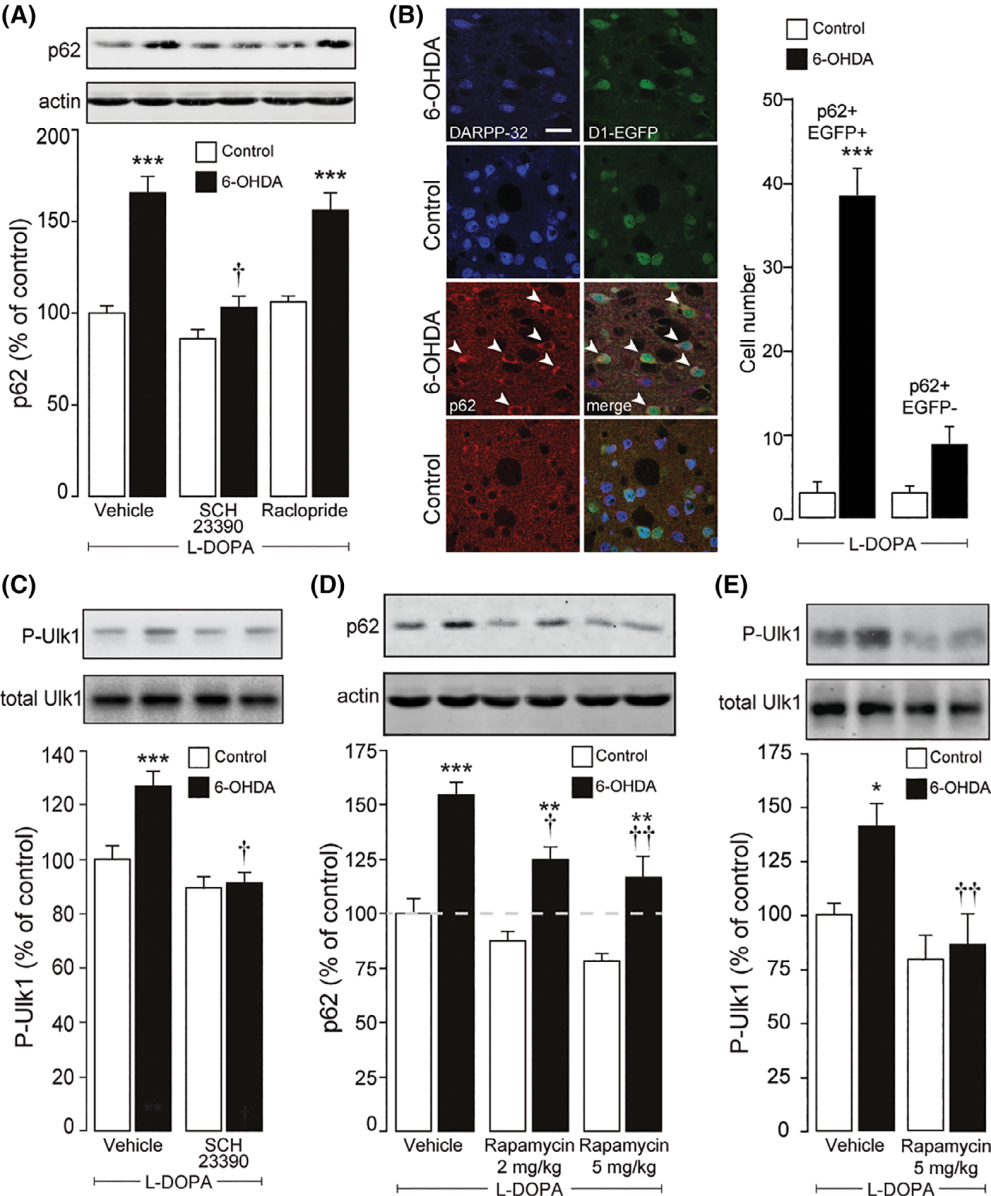
The impairment of autophagy induced by levodopa (l‐dopa) is caused by dopamine D1 receptor (D1R)‐mediated activation of mTOR complex 1 (mTORC1) in the striatal projection neurons of the direct pathway (dSPN). (**A**) Mice (n = 8–9 per group) with a unilateral 6‐hydroxydopamine (6‐OHDA) lesion received chronic (9 days) pretreatment with l‐dopa (10 mg/kg) alone (vehicle), or in combination with SCH23390 or raclopride (0.125 mg/kg and 0.25 mg/kg, respectively, administered 15 minutes before l‐dopa). The levels of p62 were measured by Western blotting 4 hours after the last drug administration in the striata contralateral (control) or ipsilateral (6‐OHDA) to the lesion. Two‐way ANOVA showed a significant effect of pretreatment (F_2,46_ = 22.59, *P* < 0.001), 6‐OHDA lesion (F_1,46_ = 70.37, *P* < 0.001), and pretreatment × 6‐OHDA lesion interaction (F_2,46_ = 7.44, *P* = 0.01). ***P < 0.001 versus control and †*P* < 0.001 versus 6‐OHDA pretreated with vehicle or raclopride, Tukey's post hoc test. (**B**) Left panels: immunofluorescence analysis of p62 and enhanced green fluorescent protein (EGFP) in a *Drd1a*‐EGFP mouse with a unilateral 6‐OHDA lesion treated for 9 days with l‐dopa (10 mg/kg) and perfused 4 hours after the last injection. DARPP‐32 immunoreactivity indicates that accumulation of p62 occurs in striatal projection neurons. Note the increased levels of p62 in the striatal projection neurons of the 6‐OHDA lesion striatum (arrowheads). Right panel: summary of data from 5 *Drd1a*‐EGFP mice showing the number of EGFP‐positive dSPN and EGFP‐negative iSPN with high levels of p62 immunoreactivity. Two‐way ANOVA showed significant effect of cell type (F_1,16_ = 52.03, *P* < 0.001), 6‐OHDA lesion (F_1,16_ = 100.8, *P* < 0.001), and cell type × 6‐OHDA lesion (F_1,16_ = 52.03, *P* < 0.001). ***P < 0.001 versus control, Sidak's multiple comparison test. (**C**) Two groups (n = 9) of mice with a unilateral 6‐OHDA lesion were treated for 9 days with l‐dopa (10 mg/kg) alone (vehicle) or l‐dopa plus SCH23390 (0.125 mg/kg), and the levels of total and phosphorylated (S757) Ulk1 (P‐Ulk1) were determined by Western blotting 4 hours after the last drug administration in the striata contralateral (control) or ipsilateral (6‐OHDA) to the lesion. Two‐way ANOVA showed a significant SCH23390 pretreatment × 6‐OHDA lesion interaction (F_1,64_ = 5.29, *P* < 0.05). ***P < 0.001 versus control and †*P* < 0.001 versus 6‐OHDA pretreated with vehicle, Tukey's post hoc test. (**D, E**) Mice with a unilateral 6‐OHDA lesion were treated for 9 days with l‐dopa (10 mg/kg) alone or in combination with rapamycin (2 or 5 mg/kg, administered 45 minutes before l‐dopa) and the levels of p62 (**D**, n = 6 per group) and total or P‐Ulk1 (**E**, n = 6–11 per group) were determined by Western blotting 4 hours after the last drug administration in the striata contralateral (control) or ipsilateral (6‐OHDA) to the lesion. (**D**) Two‐way ANOVA showed a significant effect of treatment (F_2,30_ = 11.37, *P* < 0.001), 6‐OHDA lesion (F_1,30_ = 68.86, *P* < 0.001), and no significant treatment × 6‐OHDA lesion interaction. ***P < 0.001 and **P < 0.01 versus respective control, †*P* < 0.05 and ††*P* < 0.01 versus 6‐OHDA/vehicle; Tukey's post hoc test. (**E**) Two‐way ANOVA showed a significant effect of treatment (F_1,30_ = 12.57, *P* < 0.001), 6‐OHDA lesion (F_1,30_ = 5.24, *P* < 0.05), and no significant treatment × 6‐OHDA lesion interaction. *P < 0.05 versus control, ††*P* < 0.01 versus 6‐OHDA/vehicle, Tukey's post hoc test.

As shown in Figure 2A, administration of SCH23390, a selective antagonist at D1R, abolished the increase in p62 produced by l‐dopa. In contrast, raclopride, a dopamine D2 receptor antagonist, did not modify the effect of l‐dopa. We also examined the cellular localization of p62 using *Drd1a*‐EGFP mice. In 6‐OHDA lesion striata, administration of l‐dopa produced a large increase in the number of EGFP‐positive cells, which correspond to dSPN, with high levels of p62 (3.2 ± 1.11 cells/section in the control versus 38.7 cells/section in the 6‐OHDA lesion striata) (Fig. 2B). In contrast, we did not observe any statistically significant change in the number of EGFP‐negative cells with high p62 (Fig. 2B).

We next determined whether the hyperactivation of mTORC1 produced by l‐dopa was responsible for the concomitant impairment of autophagy. We started by testing the effect of l‐dopa on the phosphorylation of Ulk1 at S757, which is regarded as a key step in the negative control exerted by mTORC1 on autophagy.^13^, ^14^, ^29^, ^30^ We found that chronic administration of l‐dopa was accompanied by augmented phosphorylation of Ulk1 in the dopamine‐depleted striatum and that this effect, similarly to the enhancement of p62, was prevented by blockade of D1R with SCH23390 (Fig. 2C). In a second group of experiments, mice with a unilateral 6‐OHDA lesion were treated with l‐dopa alone, or in combination with rapamycin (5 mg/kg), an inhibitor of mTORC1.^6^ Rapamycin reduced p62 in the 6‐OHDA lesion striatum (Fig. 2D). A comparison between the levels of p62 in control (intact) and 6‐OHDA lesion striata showed that l‐dopa was still able to produce a significant, albeit reduced, effect on p62 despite the presence of rapamycin (Fig. 2D). In addition, rapamycin abolished the increase in phosphorylation of Ulk1 produced by l‐dopa in the dopamine‐depleted striatum (Fig. 2E).

**FIG. 3:**
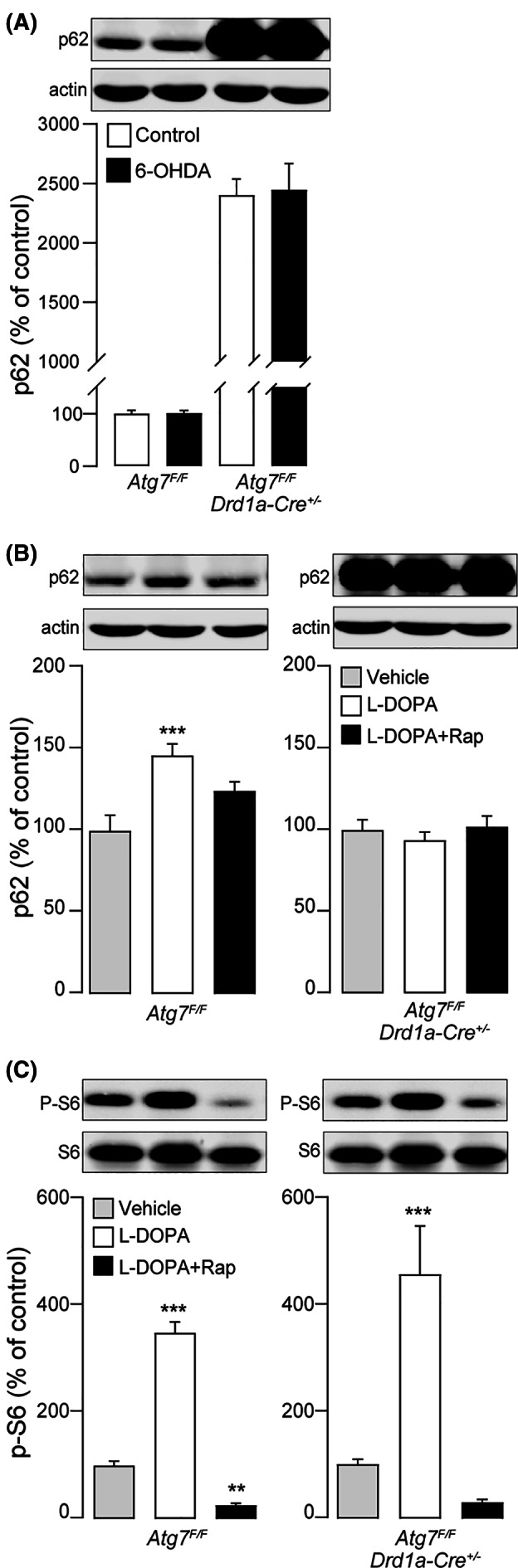
The autophagy promoting effect of rapamycin is occluded in *Atg7* ^*F/F*^ *;Drd1a‐Cre* ^*+/−*^ mice. (**A**) *Atg7* ^*F/F*^ *;Drd1a‐Cre* ^*+/−*^ and *Atg7* ^*F/F*^ mice were injected unilaterally with 6‐hydroxydopamine (6‐OHDA) and the levels of p62 was measured by Western blotting in the striata ipsilateral (control) and contralateral (6‐OHDA) to the lesion. Note the large accumulation of p62 in *Atg7* ^*F/F*^ *;Drd1a‐Cre* ^*+/−*^ mice indicative of impaired autophagy in the striatal projection neurons of the direct pathway (dSPN). 6‐OHDA did not affect striatal p62 levels (control 100.0 ± 6.3 vs. 6‐OHDA 100.7 ± 5.4 in *Atg7* ^*F/F*^ mice and control 2400 ± 137.1 vs. 6‐OHDA 2446 ± 220.2 in *Atg7* ^*F/F*^ *;Drd1a‐Cre* ^*+/−*^ mice) (n = 5–6 per group). (**B, C**) *Atg7* ^*F/F*^ *;Drd1a‐Cre* ^*+/−*^ and *Atg7* ^*F/F*^ mice with a 6‐OHDA lesion were treated with vehicle, levodopa (l‐dopa) (10 mg/kg), or l‐dopa plus rapamycin (5 mg/kg, administered 45 minutes before l‐dopa) for 9 days and the levels of p62 (**B**), total S6 and S6 phosphorylated at S240/244 (**C**) were measured by Western blotting 8 hours after the last injection. ***P < 0.001 and **P < 0.01 versus vehicle, one‐way ANOVA followed by Dunnett's multiple comparison test (n = 4–9 per group).

Finally, we tested the effect of rapamycin in *Atg7* ^*F/F*^ *;Drd1a‐Cre* ^*+/−*^ mice, which lack the gene coding for Atg7, a core Atg involved in autophagosome formation,^31^ in D1R‐expressing dSPN.^32^ In these animals, loss of Atg7 results in the constitutive impairment of autophagy, as indicated by a considerable accumulation of p62 (Fig. 3A). Moreover, in both genotypes the lesion with 6‐OHDA did not affect striatal p62 (Fig. 3A). In another experiment, the levels of p62 and phosphorylated ribosomal protein S6 were measured in the striata of 6‐OHDA lesion *Atg7* ^*F/F*^ and *Atg7* ^*F/F*^ *;Drd1a‐Cre* ^*+/−*^ mice treated with vehicle, l‐dopa (10 mg/kg), or a combination of l‐dopa and rapamycin (5 mg/kg). We found that in contrast to control *Atg7* ^*F/F*^ mice, rapamycin did not alter p62 levels in *Atg7* ^*F/F*^ *;Drd1a‐Cre* ^*+/−*^ mice (Fig. 3B). Notably, in both genotypes rapamycin retained its ability to abolish the phosphorylation of the ribosomal protein S6 on S240/244, a downstream marker of mTORC1 activation involved in the control of protein synthesis^33^, ^34^ (Fig. 3C).

**FIG. 4:**
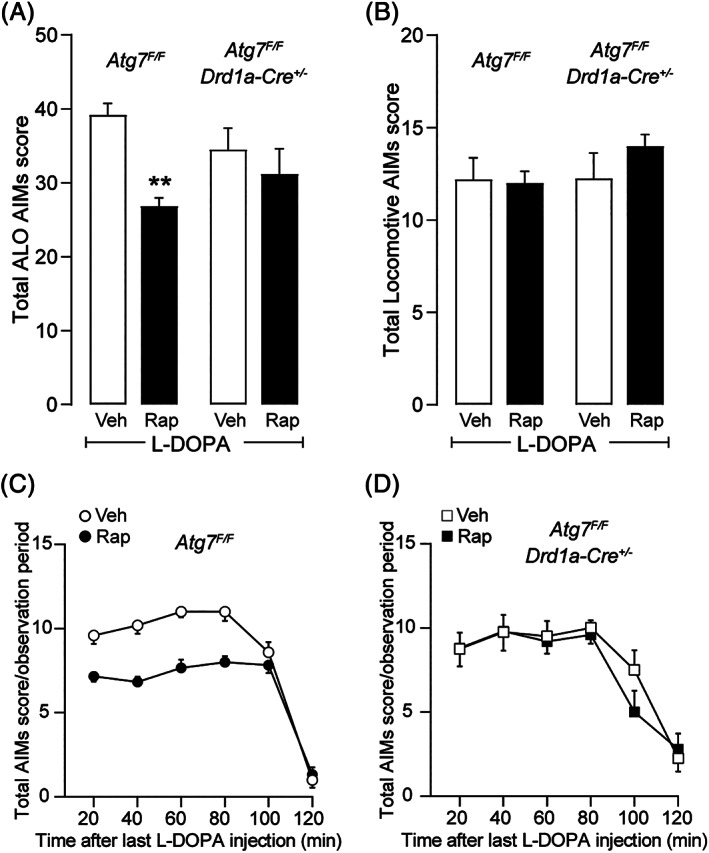
The anti‐dyskinetic action of rapamycin is prevented in *Atg7* ^*F/F*^ *;Drd1a‐Cre* ^*+/−*^ mice. *Atg7* ^*F/F*^ *;Drd1a‐Cre* ^*+/−*^ and *Atg7* ^*F/F*^ mice (n = 4–6 per group) with a unilateral 6‐hydroxydopamine (6‐OHDA) lesion were treated for 9 consecutive days with levodopa (l‐dopa) (10 mg/kg) plus vehicle or l‐dopa plus rapamycin (5 mg/kg). Abnormal involuntary movements (AIMs) were assessed for 1 minute every 20 minutes, starting immediately after the last injection. (**A**) Cumulative effect on axial, limb, orofacial (ALO) AIMs during the entire observation period (120 minutes). Two‐way ANOVA showed a significant effect of treatment (F_1,7_ = 26.62, *P* < 0.01), no effect of genotype (F_1,9_ = 0.01, *P* > 0.9), and a significant effect of genotype × treatment interaction (F_1,7_ = 8.28, *P* < 0.05). **P < 0.01 versus l‐dopa/vehicle, Sidak's multiple comparison test. (**B**) Sum of locomotive AIMs scored during the 120‐minute observation period. (**C, D**) Time course of total AIMs scored in *Atg7* ^*F/F*^ (**C**) and *Atg7* ^*F/F*^ *;Drd1a‐Cre* ^*+/−*^ mice (**D**) every 20 minutes during the 120‐minute observation period. (**C**) Two‐way ANOVA indicated a significant effect of rapamycin treatment (F_1,9_ = 69.2; *P* < 0.001), time (F_3,31_ = 108.6; *P* < 0.001), and treatment × time interaction (F_5,45_ = 6.4; *P* < 0.001). (**D**) Two‐way ANOVA indicated a significant effect of time (F_2,17_ = 25.6; *P* < 0.001).

Against this background, the ability of rapamycin to decrease LID was examined in *Atg7* ^*F/F*^ and *Atg7* ^*F/F*^ *;Drd1a‐Cre* ^*+/−*^ mice. We found that l‐dopa produced a similar dyskinetic response in the two genotypes (Fig. 4A,C,D). In line with previous work,^6^, ^17^ administration of rapamycin to *Atg7* ^*F/F*^ mice reduced LID. The counteracting effect of rapamycin was limited to axial, limb, and orofacial AIMs, which are a more reliable indicator of dyskinesia (Fig. 4A). No effect was observed on locomotive AIMs, which are instead regarded as a marker of motor impairment^35^ (Fig. 4B). In contrast, the anti‐dyskinetic action of rapamycin was abolished in *Atg7F/F;Drd1a‐Cre^+/−^ mice* (Fig. 4A,D).

## Discussion

This study shows that in mouse and NHP models of PD, dyskinesia, a serious motor disorder caused by administration of standard anti‐parkinsonian medications, is associated with molecular changes linked to impaired autophagy. We also show that a considerable proportion of the anti‐dyskinetic action of rapamycin depends on its ability to promote autophagy.

In PD, the loss of dopaminergic input to the dorsal striatum leads to the sensitization of D1R,^3^, ^4^, ^5^ which confers on l‐dopa the ability to activate mTORC1 signaling in dSPN.^6^, ^7^ The ability of rapamycin, an inhibitor of mTORC1, to counteract dyskinesia has been related to its action on mTORC1 downstream targets involved in the control of protein synthesis.^6^, ^36^ However, mTORC1 regulates multiple substrates, including signaling components, such as Ulk1, implicated in the control of autophagy. Ulk1 forms a complex with Atg13, FIP200 (focal adhesion kinase family interacting protein of 200 kDa), and Atg10, necessary for autophagosome formation.^13^, ^14^, ^30^ Association of activated mTORC1 with the Ulk1 complex leads to direct phosphorylation of Ulk1 and Atg13 by mTOR and inhibition of the autophagy promoting kinase activity of the Ulk1 complex.^13^, ^14^, ^30^ The resulting block of p62 degradation in the autophagosome leads to p62 accumulation, a standard marker of autophagy impairment.^15^, ^16^

In line with the abnormal activation of mTORC1 observed in LID, we found that this condition is associated with mTORC1‐mediated phosphorylation of Ulk1 at the inhibitory site S757 and with increased levels of p62. We also found that these effects are abolished by blockade of D1R, but not D2R, and that in *Drd1a*‐EGFP mice, higher levels of p62 immunoreactivity, induced by l‐dopa in the dopamine depleted striatum, are restricted to EGFP‐positive cells. These observations, together with previous studies showing the selective activation of mTORC1 signaling in D1R‐expressing striatal neurons,^6^, ^37^ indicate that the impairment of autophagy occurs in a neuronal subpopulation corresponding to the dSPN.

It has been shown that the development of LID depends on combined dysregulated transmission in dSPN^38^, ^39^ and in the D2R‐expressing projection neurons of the indirect pathway (iSPN).^40^, ^41^ Thus, blockade of D2R with raclopride or chemogenetic activation of iSPN (which generate an analogous functional response) are also able to reduce LID in a mouse model of PD.^40^, ^41^ In view of these findings, the inability of raclopride to reduce the accumulation of p62 associated with LID suggests that the anti‐dyskinetic action of this drug occurs through a parallel mechanism, which circumvents the effects of reduced autophagy in dSPN.

We show that administration of rapamycin reduces the accumulation of p62 caused by l‐dopa in the 6‐OHDA lesion striatum. Our results also indicate that this effect is not complete, since we still observe a significant difference compared to the respective control striatum treated with rapamycin. This partial reduction of p62 may represent an advantage in clinical settings since it suggests a normalization rather than an excessive activation of autophagy, which in the long term might cause undesired side effects.

To test the hypothesis that the dyskinetic action of rapamycin depends on its autophagy promoting properties, we used *Atg7* ^*F/F*^ *;Drd1a‐Cre* ^*+/−*^ mice. In this transgenic mouse line, autophagy is suppressed in dSPN, as indicated by abnormal levels of striatal p62. A recent study examined the phenotype of *Atg7* ^*F/F*^ *;Drd1a‐Cre* ^*+/−*^ mice and did not report modifications of locomotor behavior in novel environments.^32^ Notably, the same study also showed that knockout of Atg7 in dSPN reduced dendritic spine density. This effect is reminiscent of the increase in spine pruning observed in dSPN following dopamine depletion^42^ and might concur to worsen the effects of 6‐OHDA. However, it should be mentioned that the analysis of locomotive AIMs, which are an index of motor impairment,^43^ did not show any difference between 6‐OHDA lesion *Atg7* ^*F/F*^ *;Drd1a‐Cre* ^*+/−*^ and control *Atg7* ^*F/F*^ mice.

Rapamycin failed to promote autophagy in *Atg7* ^*F/F*^ *;Drd1a‐Cre* ^*+/−*^ mice as indicated by the lack of effect on p62 accumulation, but maintained its ability to decrease mTORC1‐mediated phosphorylation of the ribosomal protein S6, a downstream effector involved in the regulation of protein synthesis.^33^, ^34^ As expected, rapamycin reduced the dyskinetic response produced by administration of l‐dopa in *Atg7* ^*F/F*^ mice. Although substantial, this effect was more moderate than that observed in a previous study in the mouse^6^ and more in line with a recent study performed in the rat.^8^ This difference may be related to the mouse strain utilized in this experiment, for example, *Atg7* ^*F/F*^ mice obtained from crossing *Atg7* ^*F/F*^ *;Drd1a‐Cre* ^*+/−*^ with *Atg7* ^*F/F*^ mice. Importantly, and in line with the involvement of dysregulated autophagy in LID, the anti‐dyskinetic effect of rapamycin was occluded in *Atg7* ^*F/F*^ *;Drd1a‐Cre* ^*+/−*^ mice. Combined with the pharmacological experiments and the cellular localization of elevated p62 in *Drd1a*‐EGFP mice, these results indicate that rapamycin reduces LID by promoting autophagy in dSPN.

The mechanisms at the basis of the anti‐dyskinetic properties of rapamycin remain to be determined. LID has been proposed to depend on defective synaptic downscaling, manifested as loss of depotentiation at corticostriatal synapses.^44^, ^45^ Hyperactivation of mTORC1 leading to defective autophagy may produce this condition, since autophagy has been involved in the degradation of glutamate AMPA receptors and in the generation of long‐term depression.^46^ It should also be noted that the constitutive impairment of autophagy caused by inactivation of Atg7 in dSPN does not enhance the dyskinetic response to l‐dopa. Whereas it is possible that the severity of LID displayed by the mouse model employed in this study precludes the exacerbation of AIMs, further studies will be necessary to determine the impact of dysregulated autophagy per se on the development of LID in the absence of rapamycin. In this context, it is interesting that LID has been also associated with the D1R‐mediated impairment of the ubiquitin‐proteasome system,^47^, ^48^ the other crucial protein degradation pathway in eukaryotes.^49^ Therefore, it appears that LID is accompanied by compromised activity of the two major catabolic systems in dSPN. The relative contribution of these pathways to the development and expression of dyskinesia and their possible crosstalk remain to be characterized.

Autophagy promoting agents, including mTOR inhibitors such as rapamycin, are regarded as a potential therapeutic strategy against cancer, diabetes, and neurodegenerative disorders.^50^, ^51^, ^52^ The present results indicate that these drugs may represent a promising avenue also for the management of dysfunctional dopamine transmission in LID.

## Conflict of interest

EB is a director and a shareholder of Motac Neuroscience Ltd. The other authors declare no conflict of interest.

## Authors’ Roles

MF, GS, AP, LU, CP, and MA performed and analyzed Western blot, immunofluorescence, and mRNA analyses in wild‐type mice. CP, OJL, AB, and ES designed, performed, and analyzed biochemical and behavioral analyses in *Atg7^*F/F*^;Drd1a‐Cre* ^*+/−*^ and *Atg7* ^*F/F*^ mice. BD, QL, and EB conceived, designed, performed, and analyzed the experiments in non‐human primates. MF, MA, DS, EB, AB, ES, PN, and GF conceived the experiments and provided essential reagents and equipment. GF directed the study and wrote the manuscript.

## Funding sources

This work was supported by the Swedish Research Council (GF, AB, and PN), the Swedish Brain Foundation (GF and PN), and the Swedish Parkinson Foundation (GF). MF and CP were supported by the Karolinska Institutet/National Institutes of Health PhD program in Neuroscience. DS is supported by NIH NINDS R01 NS095435, and NIDA R01 DA07418, and the Simons and JPB Foundations. AB is supported by the Strategic Research Area in Neuroscience at Karolinska Institutet (StratNeuro), and the Åhlens and Magnus Bergvalls Foundations. PN is supported by the Hållstens Foundation.
